# Identification of ARHGAP9 as a Key Diagnostic Marker for Abdominal Aortic Aneurysm by Multiomics and Experimental Validation

**DOI:** 10.1155/humu/7230083

**Published:** 2025-09-16

**Authors:** Zhe Peng, Kun Li, Shile Wu, Baozhang Chen, Xiaonan Wang, Liang Chen, Xinsheng Wang, Hao Zhang, Biao Wu

**Affiliations:** ^1^Department of General Surgery, Qinghai Province People's Hospital, Xining, Qinghai, China; ^2^Department of Vascular Surgery, Changhai Hospital, Shanghai, China; ^3^Department of Vascular Surgery, 967 Hospital of the Joint Logistics Support Force of PLA, Dalian, Liaoning, China

**Keywords:** abdominal aortic aneurysm, ARHGAP9, biomarker, machine learning

## Abstract

Abdominal aortic aneurysm (AAA) is a serious vascular condition that significantly endangers the lives of patients. Although there have been improvements in early detection and treatment methods, considerable challenges persist regarding the timely identification and evaluation of risk associated with this disease. Therefore, there is an immediate requirement for novel biomarkers that can enhance the early diagnosis and risk evaluation of AAA, thus allowing for more accurate and individualized medical interventions. In this study, we identified key diagnostic markers for AAA using various machine learning algorithms, and we explored the functions of these genes in AAA through gene enrichment analysis. A diagnostic model for AAA was constructed based on multiple machine learning algorithms, with the random forest algorithm highlighting the central role of ARHGAP9. In vitro experiments confirmed the influence of ARHGAP9 on vascular smooth muscle cells (VSMCs). Our findings indicate that the key genes identified are associated with the immune microenvironment and metabolism in AAA samples. The validated diagnostic model exhibited excellent predictive performance. Knockdown of ARHGAP9 significantly inhibited the proliferative capacity of VSMCs. In conclusion, our results suggest that ARHGAP9 may serve as a diagnostic and therapeutic marker for AAA.

## 1. Introduction

Abdominal aortic aneurysm (AAA) is a serious vascular condition marked by the localized expansion of the abdominal aorta, especially common in older males. This ailment is notably more prevalent in Western nations and represents a considerable risk to the lives of affected individuals [1–3]. An AAA rupture can often be fatal for asymptomatic patients, with a mortality rate reaching at least 4.5 fatalities per 1000 people [4]. The development of this condition is multifaceted, involving the breakdown of elastic fibers, the programmed cell death of smooth muscle cells, inflammatory responses, and a complex interaction of genetic predispositions and environmental influences. These alterations contribute to the gradual deterioration of the vascular wall structure and changes in hemodynamics, elevating the likelihood of arterial rupture and subsequent life-threatening situations [5, 6]. Although advancements in early detection and treatment have been made—such as the broad adoption of ultrasound imaging and enhancements in surgical methods—significant hurdles persist, particularly regarding the early detection of the disease and assessment of risks. Additionally, current imaging techniques largely depend on ultrasound or CT scans, which, despite being highly reliable for diagnosis, do not completely capture microscopic changes within the vascular wall or forecast the aneurysm's growth trajectory [7]. Research into the disease at a molecular level during its early stages remains underdeveloped, and there is a deficiency of accurate biomarkers to direct personalized treatment strategies. In conclusion, there is a critical necessity for innovative biomarkers and detection technologies to facilitate early diagnosis and risk evaluation of AAA, thus enabling more precise and individualized medical interventions, which could lower rupture rates and enhance patient outcomes.

AAA is a potentially fatal condition characterized by a high risk of rupture. Currently, there is a significant lack of effective biomarkers for predicting the progression and rupture risk of AAA. However, recent years have seen progress in the exploration of new biomarkers. Research indicates that C-C chemokine receptor 2 (CCR2) may serve as both a therapeutic and diagnostic marker for AAA. In rat models, CCR2-targeted positron emission tomography imaging agents have demonstrated the potential to predict AAA rupture [8]. Furthermore, studies have demonstrated that miR-3154 exacerbates the phenotypic switching of vascular smooth muscle cells (VSMCs) and the development of AAA in a dose-dependent manner, both in vivo and in vitro. Therefore, miR-3154 may serve as a promising therapeutic target and prognostic biomarker for the treatment of AAA [9]. Additionally, proteomic analyses have uncovered several biomarkers associated with AAA. For instance, studies indicate that glutathione, glycine, and serine are linked to the volume growth rate of AAA, suggesting that these metabolites could serve as potential markers for monitoring AAA progression [10]. Moreover, research has identified 39 differentially expressed proteins in the plasma of patients with AAA, including legumain (LGMN), which is significantly elevated in these patients [11]. Furthermore, research has demonstrated that the activation of the nuclear receptor pregnane X receptor (PXR) can mitigate the development and progression of AAA by inhibiting oxidative stress [12]. Additionally, studies employing single-cell sequencing and machine learning have identified macrophage-associated genes, such as THBS1, which are upregulated in AAA and may serve as novel therapeutic targets [13]. Although these studies have opened new avenues for investigating biomarkers of AAA, further clinical validation and large-scale studies are essential to confirm the diagnostic and predictive value of these markers. Through ongoing research and exploration, it is anticipated that more effective diagnostic and therapeutic strategies for AAA will be developed in the future.

In recent years, the development of high-throughput sequencing and big data analysis has significantly increased the application of multiomics approaches in tumor and inflammatory disease research [14–17]. Multiomics technology integrates various levels of data, including genomics, transcriptomics, proteomics, and metabolomics, enabling a comprehensive and systematic elucidation of the molecular mechanisms underlying biological processes (BPs). Compared to single-omics, multiomics integration mitigates data limitations, enhances the efficiency and accuracy of disease biomarker screening, and is particularly well suited for the investigation of complex diseases. This study provides a comprehensive analysis of key diagnostic genes associated with AAA through the application of multiple machine learning algorithms. Functional analysis revealed the significant roles of these genes in AAA. Additionally, we constructed and validated a diagnostic model, ultimately identifying ARHGAP9 as a key diagnostic gene. Furthermore, we confirmed its role in AAA through in vitro experiments.

## 2. Materials and Methods

### 2.1. Sample Collection

This study incorporated three datasets related to AAA: GSE119717, GSE57691, and GSE47472. GSE119717 consists of 60 AAA samples and 58 normal samples, GSE57691 includes 49 AAA samples and 10 normal samples, while GSE47472 comprises eight AAA samples and 14 normal samples.

### 2.2. Weighted Gene Coexpression Network Analysis (WGCNA)

WGCNA was utilized to classify genes according to their expression similarities, which enabled the exploration of relationships between various gene clusters and clinical traits. This widely recognized method assists in examining the ties between clinical characteristics and gene expressions. The focus of this study was on patients undergoing radiotherapy within the AAA cohort. A coexpression network was specifically constructed for the radio-sensitive (RS) and radio-resistant (RR) groups using the R package “WGCNA.” To assess gene interactions in relation to a scale-free distribution, the optimal soft threshold was determined. Following this, a dendrogram was created to represent gene adjacency and similarity measures. To improve module separation, a dynamic tree cutting algorithm was applied to combine closely related modules, with each unique color in the dendrogram corresponding to a distinct module of genes that display similar expression patterns. The relationship between each gene module and sample characteristics was evaluated using Pearson's correlation, resulting in the identification of several modules with higher absolute correlation values for further analysis [18].

### 2.3. Functional Analysis

In order to further investigate the roles of the diagnostic genes associated with AAA that we have identified, we performed a functional enrichment analysis on the collected data. The Gene Ontology (GO) serves as a prominent instrument for the annotation of gene functions, specifically focusing on molecular function (MF), BP, and cellular component (CC). Additionally, KEGG enrichment analysis provides a valuable approach for examining gene functions and associated advanced genomic information. To enhance our understanding of the oncogenic functions of the target genes, we utilized the ClusterProfiler package within R to analyze the GO functions of potential mRNAs and to enrich KEGG pathways [19].

### 2.4. Molecular Docking

Molecular docking was conducted via the CB-Dock2 website [20]. The molecular structure of ARHGAP9 was retrieved from the Protein Data Bank database, while the 3D structure of the drug was obtained from the PubChem database.

### 2.5. Constructing Diagnostic Models Using Multiple Machine Learning Algorithms

In order to create a diagnostic model for AAA that shows reliable accuracy and performance, different configurations of machine learning algorithms were utilized. The training of the model occurred with the GSE119717 dataset, whereas validation involved the GSE57691 and GSE47472 datasets. Each combination of algorithms was assessed using the area under the curve (AUC) metric, and the one with the highest average AUC was chosen as the best model.

### 2.6. Analysis of Immune Infiltration Levels

The R package GSVA was utilized to assess the infiltration of immune cells in samples of AAA through the single-sample gene set enrichment analysis (ssGSEA) approach [21, 22]. Additionally, the ggstatsplot package was used to create a heat map that depicts patterns of immune infiltration. To explore the associations between quantitative variables that were not normally distributed, Spearman's correlation analysis was conducted.

### 2.7. Quantitative Reverse Transcription PCR (qRT-PCR)

Total RNA was extracted using TRIzol reagent (Thermo Fisher, Cat. 15596018CN, Carlsbad, CA, United States). The qRT-PCR was conducted utilizing FastStart Universal SYBR Green Master (D7260, Beyotime, China) on a LightCycler 480 PCR System (Thermo Fisher Scientific, Waltham, MA, United States). For the reaction, cDNA was employed at a total volume of 20 *μ*L, which included 2 *μ*L of cDNA template, 0.5 *μ*L each of forward and reverse primers, 10 *μ*L of PCR mixture, and the necessary volume of water. The PCR process was carried out under the following cycling parameters: an initial denaturation step at 95°C for 30 s, followed by 45 cycles of 94°C for 15 s, 56°C for 30 s, and 72°C for 20 s. Each sample was analyzed in triplicate. The threshold cycle (CT) values were determined using the 2^−ΔΔCT^ method and normalized to ACTB levels [23]. The primer sequences for the target gene are as follows: ARHGAP9 (forward: CAGAGCAGTGCCTCTCTC, reverse: CTGCTGGGTCAGATGTCTC), *β*-actin (forward: GAGACCTTCAACACCCCAGC, reverse: ATGTCACGCACGATTTCCC), IL-6 (forward: AGACAGCCACTCACCTCTTCAG, reverse: TTCTGCCAGTGCCTCTTTGCTG), and IL-8 (forward: GAGAGTGATTGAGAGTGGACCAC, reverse: CACAACCCTCTGCACCCAGTTT).

### 2.8. Cell Culture

VSMCs sourced from Jennio Biotech (Guangzhou, China) were grown in a medium enriched with smooth muscle growth factors (Cascade Biologics, Karlsruhe, BW, Germany), along with 10% fetal bovine serum (FBS; Gibco-BRL, Grand Island, NY, United States) and antibiotics consisting of penicillin and streptomycin. The cells underwent incubation at 37°C in a humidified atmosphere with 5% CO_2_ [24].

### 2.9. CCK-8

The cell viability was assessed using the CCK-8 assay (Cat. C0038, Beyotime, Shanghai, China). In brief, differently treated cells were cultured in 96-well plates (NEST, China) at a density of 1 × 10^3^ cells per well. At designated time points, CCK-8 solution was added. The optical density (OD) at 450 nm was measured with a microplate reader following a 2-h incubation of the cells at 37°C [23].

### 2.10. Statistical Analysis

The Wilcoxon test was employed to calculate statistical differences between the two groups, with a two-tailed *p* value of less than 0.05 considered to indicate statistical significance.

## 3. Results

### 3.1. Identification of Key Diagnostic Genes for AAA Based on WGCNA

**Figure 1:**
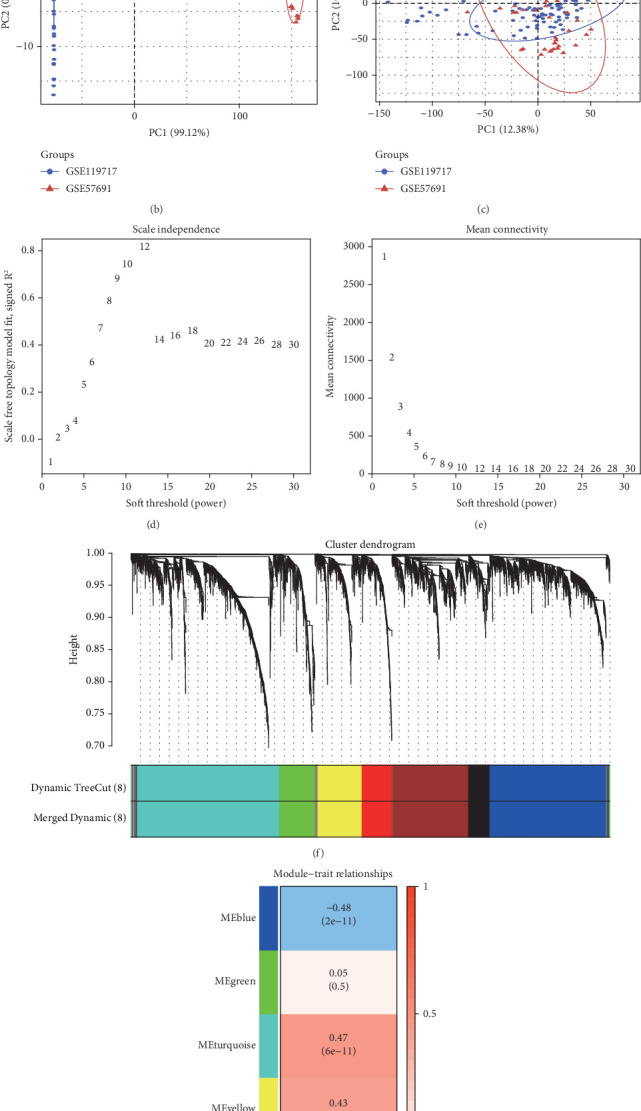
WGCNA identified 100 key genes. (a) The data, standardized, is illustrated as a box plot. (b) PCA outcomes before the adjustment for batch effects across different datasets. (c) PCA results post the removal of batch effects among the separate datasets. (d, e) The ideal power for soft-thresholding was determined to be 12. (f) A weighted coexpression network was built utilizing the chosen power values. (g) A heat map was generated to represent the correlations within trait modules.

To identify biomarkers related to diagnosing AAA, we sourced data from two separate datasets (GSE119717 and GSE57691) available in the GEO database, which encompassed 109 samples from patients diagnosed with AAA and 68 samples from healthy controls. Initially, we normalized and merged these high-throughput sequencing datasets to create a comprehensive dataset for further analysis. Next, we conducted principal component analysis, illustrating the results before and after the removal of batch effects, which validated the successful integration of the datasets for additional examination (Figures 1a, 1b, and 1c). In order to identify diagnostic-related genes that might influence AAA, we performed WGCNA on the integrated dataset. To adhere to scale-free topology principles, we modified the weight settings of the adjacency matrix and identified 12 as the best power value (Figure 1d,e). As a result, we built a weighted coexpression network, organizing all genes into eight separate modules (Figure 1f). Employing the Pearson correlation method, we assessed the correlation coefficients and corresponding *p* values between module eigengenes and specific traits. The findings indicated that the blue module showed the most pronounced negative correlation, with a correlation coefficient of −0.48, while the turquoise module indicated the highest positive correlation, with a correlation coefficient of 0.47 (Figure 1g). We then selected the Top 50 genes from these two modules for further investigation.

### 3.2. Expression Differences of Key Genes in AAA

**Figure 2:**
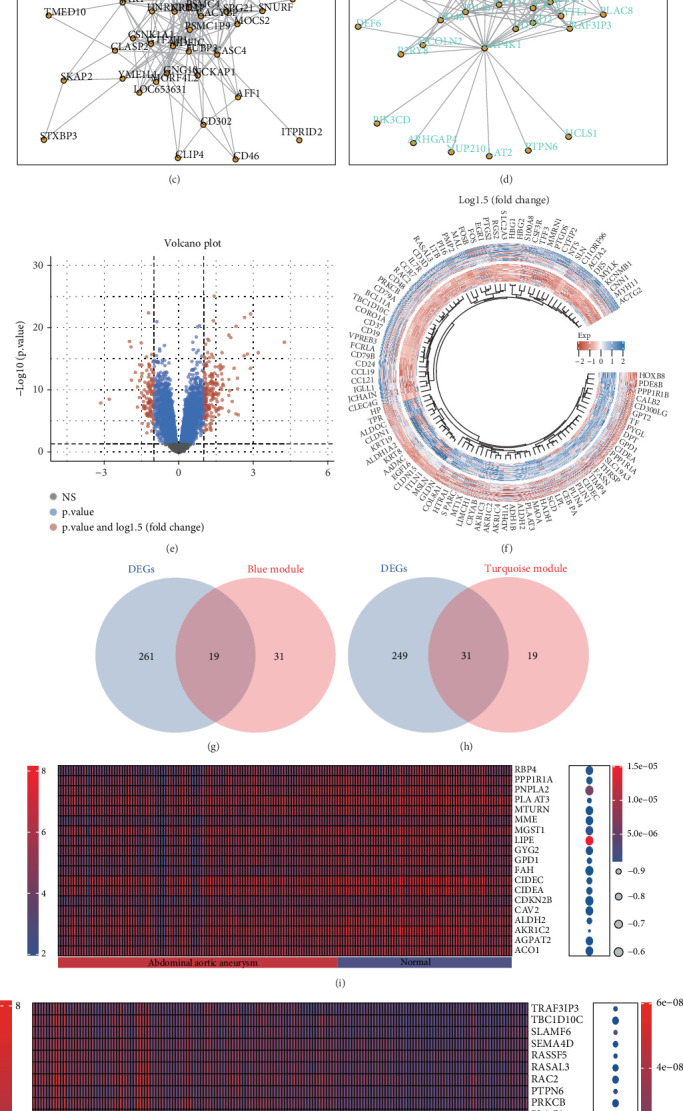
We identified 50 differentially expressed genes. (a, b) Scatter plot of specific traits associated with module genes. (c, d) Interaction network diagram of module genes. (e, f) Differential analysis heat map and volcano plot. (g, h) Intersection of differentially expressed genes and key module genes. (i, j) Heatmap of differentially expressed genes.

In the WGCNA, we identified genes in the blue and turquoise modules as key diagnostic markers for AAA. We first presented scatter plots illustrating the associations between the genes in these modules and specific traits of AAA (Figure 2a,b). Additionally, we displayed interaction network diagrams of the Top 50 genes from both modules (Figure 2c,d). Subsequently, we conducted a differential analysis by merging the GSE119717 and GSE57691 datasets, applying screening criteria of a *p* value less than 0.05 and a LogFC greater than 1.5 or less than −1.5, resulting in the identification of 280 differentially expressed genes (Figure 2e,f). Furthermore, we illustrated the intersections of differentially expressed genes within the blue and turquoise modules using Venn diagrams. The blue module contained 19 differentially expressed genes, while the turquoise module encompassed a total of 31 differentially expressed genes (Figure 2g,h). Finally, the expression differences of the aforementioned screened genes were displayed through a heat map (Figure 2i,j).

### 3.3. Functional Analysis of Key Genes

**Figure 3:**
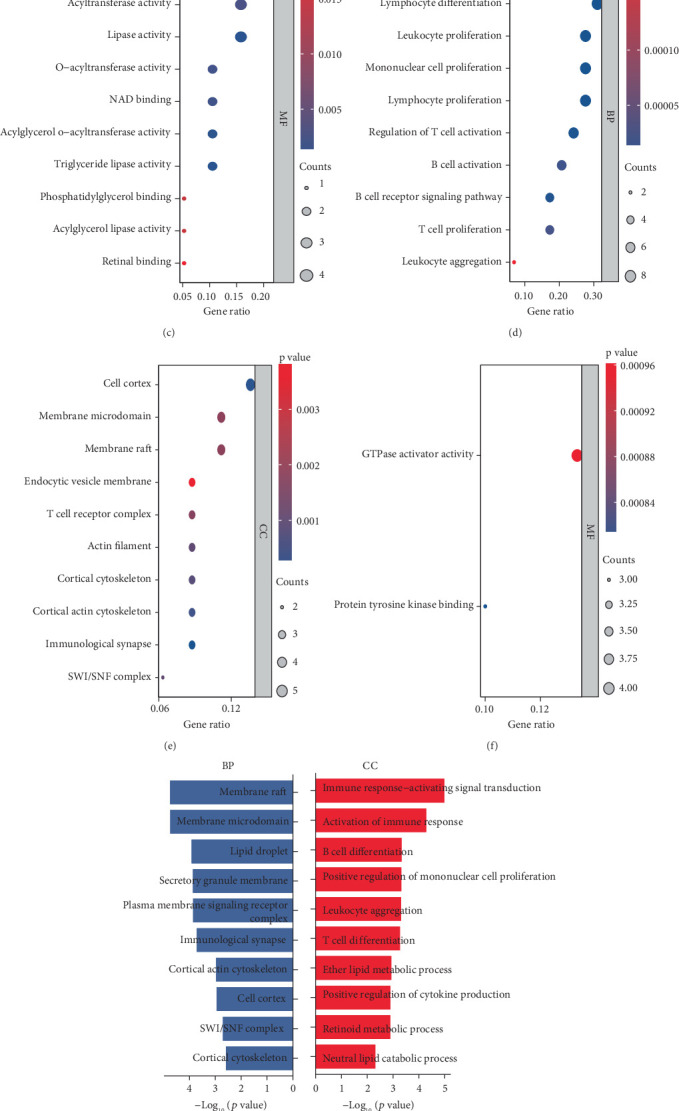
Functional analysis of key genes using KEGG and GO. (a–c) Functional analysis of key genes in the blue module using GO analysis. (d–f) Functional analysis of key genes in the turquoise module using GO analysis. (g) GO analysis of the functional roles of key genes in the blue and turquoise modules. (h) KEGG analysis of the functional roles of key genes in the blue and turquoise modules.

To explore the functions of the identified genes, we conducted KEGG and GO analyses. Initially, we performed GO analysis on the 19 key genes within the blue module. The results indicated that these key genes are associated with the primary alcohol metabolic process, glycerolipid metabolic process, hormone metabolic process, secretory granule membrane, lipid droplet, membrane microdomain, carboxylic ester hydrolase activity, acyltransferase activity, and lipase activity (Figures 3a, 3b, and 3c). Subsequently, we conducted GO analysis on the 31 key genes identified within the turquoise module. The results of this analysis indicated that these key genes are associated with various BPs, including mononuclear cell differentiation, lymphocyte differentiation, leukocyte proliferation, and CCs, such as the cell cortex, membrane microdomain, and membrane raft. Additionally, the analysis highlighted their involvement in GTPase activator activity and protein tyrosine kinase binding (Figures 3d, 3e, and 3f). Afterward, we combined all 50 genes for analyses utilizing GO and KEGG. Results from the GO analysis demonstrated that these genes are linked to membrane rafts, membrane microdomains, the cortical actin cytoskeleton, immune response-activating signal transduction, the activation of immune responses, and B cell differentiation (Figure 3g). The KEGG findings illustrated their connection to the B cell receptor signaling pathway, natural killer cell-mediated cytotoxicity, and glycerolipid metabolism (Figure 3h). In conclusion, we determine that these essential genes are mainly associated with the immune microenvironment and the metabolism related to AAA.

### 3.4. Machine Learning Algorithms Construct Diagnostic Models

**Figure 4:**
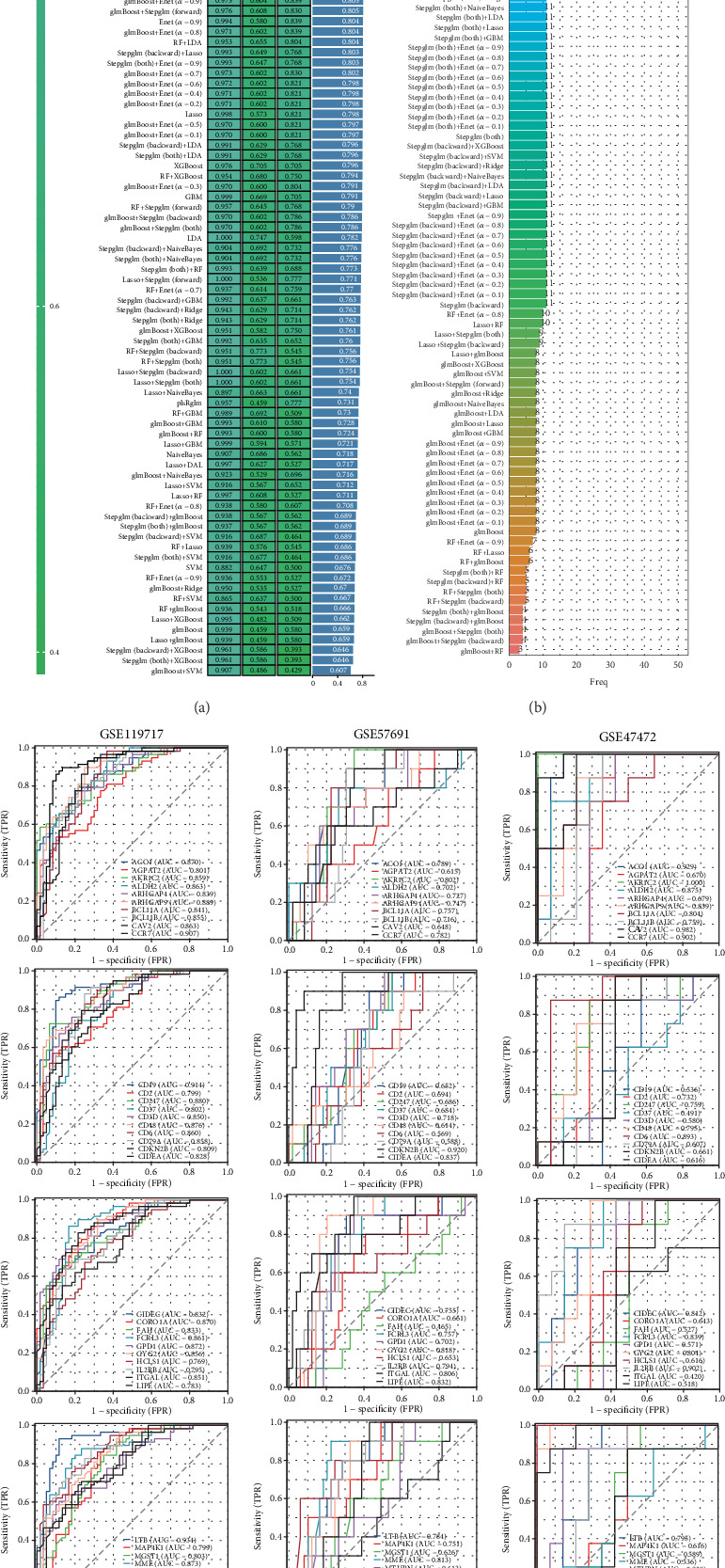
Construct a diagnostic model. (a) AUC values of different algorithms. (b) The number of genes included by different algorithms. (c–e) The predictive value of key genes in different datasets for the diagnosis of AAA.

To explore the significance of the identified key genes in diagnosing AAA, multiple machine learning techniques were utilized to assess their viability as predictive biomarkers for this condition. Our research aimed at building diagnostic models with three datasets: the GSE119717 dataset for training purposes, while the GSE57691 and GSE47472 datasets were used for validation. Among the different combinations of algorithms evaluated, the Ridge, Enet[alpha = 0.3], Enet[alpha = 0.1], and Enet[alpha = 0.2] methods exhibited the most effective performance in developing the models. The diagnostic frameworks created with these four algorithms attained an average AUC of 0.878 across all five datasets, indicating strong predictive performance (Figure 4a). Nevertheless, the count of genes utilized by each algorithm differs slightly. The Ridge algorithm incorporates all genes, while the Enet[alpha = 0.3] algorithm comprises 46 genes, the Enet[alpha = 0.1] algorithm contains 49 genes, and the Enet[alpha = 0.2] algorithm consists of 47 genes (Figure 4b). Finally, we demonstrated the independent predictive capability of these 50 genes through ROC curves (Figures 4c, 4d, and 4e).

### 3.5. The Random Forest Algorithm Further Screens Key Genes

**Figure 5:**
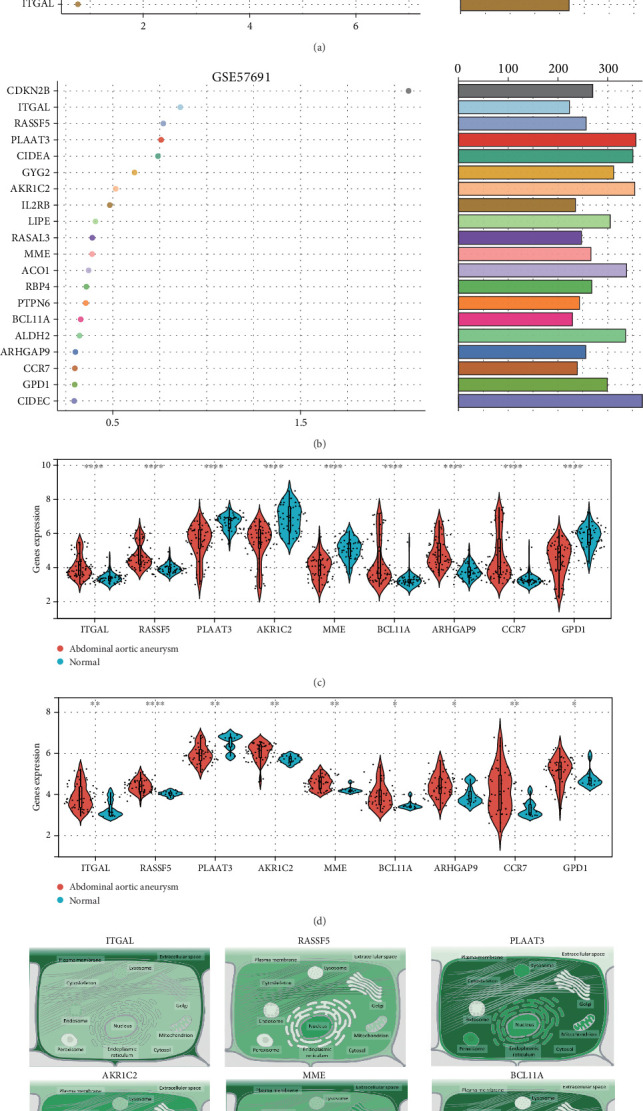
The random forest algorithm further screens key genes. (a, b) Random forest algorithm identifies the Top 20 key genes. (c, d) Expression analysis of key genes. (e) Molecular localization analysis of key genes.

In order to minimize the number of genes examined and pinpoint the most essential diagnostic genes, we applied the random forest algorithm once more to refine the selection of the 50 previously identified genes. Within the GSE119717 dataset, the following genes were recognized as critical: LTB, CCR7, CD19, PRKCB, FCRL3, MME, RASSF5, CAV2, ARHGAP9, TBC1D10C, CD79A, BCL11A, AKR1C2, TRAF3IP3, CD247, GPD1, PLAC8, PLAAT3, CD48, and ITGAL. Similarly, in the GSE57691 dataset, the key genes identified included CDKN2B, ITGAL, RASSF5, PLAAT3, CIDEA, GYG2, AKR1C2, IL2RB, LIPE, RASAL3, MME, ACO1, RBP4, PTPN6, BCL11A, ALDH2, ARHGAP9, CCR7, GPD1, and CIDEC (Figure 5a,b). Subsequently, we conducted further analysis on the nine genes common to both datasets. Initially, we visualized the expression differences of these nine genes in the GSE119717 and GSE57691 datasets using violin plots (Figure 5c,d). Finally, we analyzed the localization of these nine genes through the GeneCard database (Figure 5e).

### 3.6. Analysis of Immune Cell Infiltration

**Figure 6:**
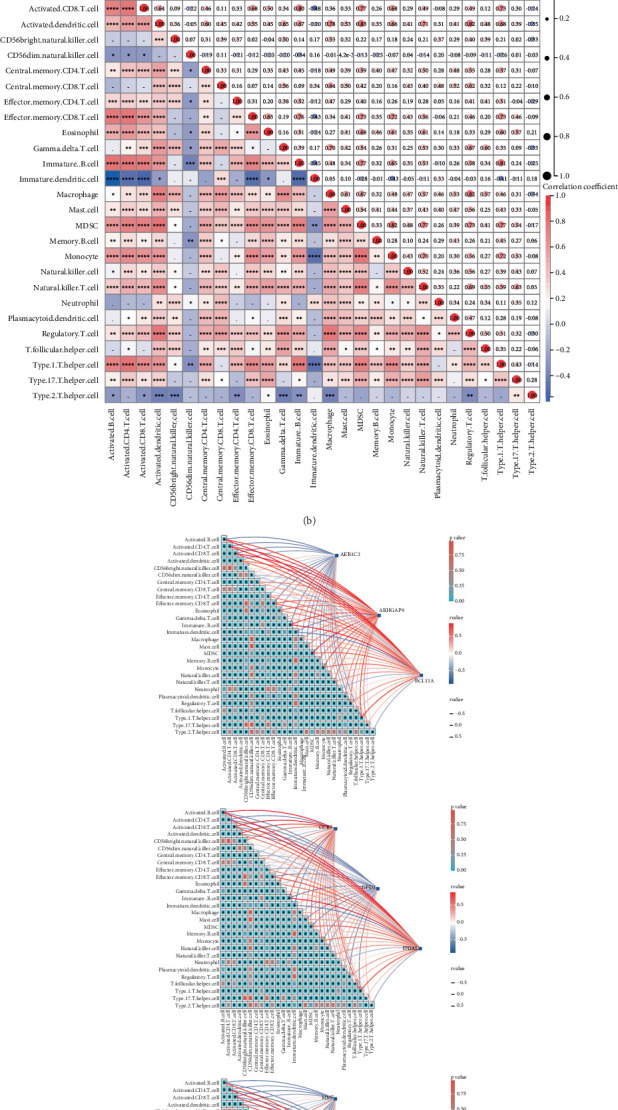
Immune analysis of key genes in AAA. (a) Analysis of immune cell infiltration levels in AAA and normal samples. (b) Correlation analysis of different immune cell infiltration levels. (c) Correlation analysis between the expression of different genes and the level of immune cell infiltration.

Recent research indicates that the infiltration of immune cells is a significant factor in the progression of AAA. In our investigation, we examined the link between the traits of AAA and immune cell infiltration. Building on the findings of Pornpimol et al. [25], who discovered 28 genes that serve as markers for different immune cells, we employed ssGSEA to evaluate the immune cell scores of patients according to this particular gene set. Initially, we assessed immune cell infiltration in both AAA samples and normal samples, discovering that most immune cell infiltration levels were elevated in AAA (Figure 6). Following this, we created heatmaps to illustrate the infiltration levels of 28 distinct immune cell types, which exhibited positive correlations among the majority of immune cell infiltration levels (Figure 6). Further analysis involved investigating the connections between the genes AKR1C2, ARHGAP9, BCL11A, CCR7, GPD1, ITGAL, MME, PLAAT3, and RASSF5 and the various immune cell types, resulting in additional heatmaps (Figure 6). We observed that AKR1C2, GPD1, MME, and PLAAT3 had negative correlations with immune cell infiltration levels, whereas the remaining six genes displayed positive correlations with these levels.

### 3.7. ARHGAP9 Is a Key Diagnostic Gene for AAA

**Figure 7:**
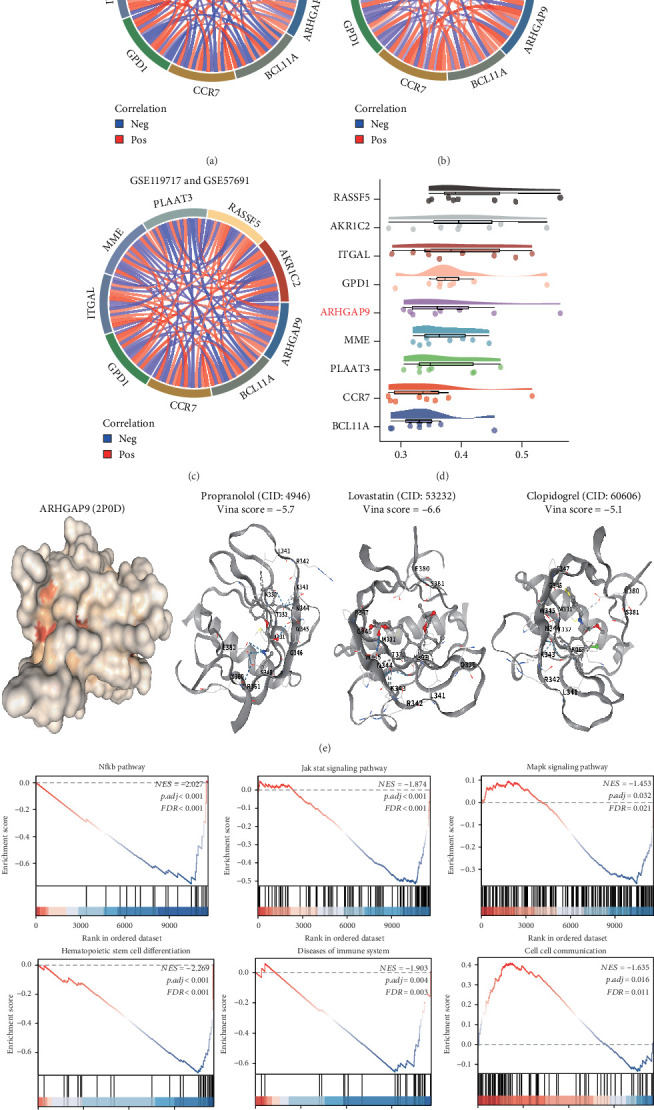
ARHGAP9 can serve as a diagnostic and therapeutic target for AAA. (a–c) Correlation analysis of key genes. (d) Ranking the importance of key genes. (e) Molecular docking of ARHGAP9 with drugs. (f) Functional analysis of ARHGAP9.

Among the nine identified genes, we analyzed their correlations using the GSE119717 and GSE57691 datasets. Our results indicate that the majority of the genes exhibit positive correlations, while a minority show negative correlations (Figures 7a, 7b, and 7c). Subsequently, we conducted a friend analysis to rank the importance of these nine genes based on gene similarity (Figure 7d). Our previous analysis revealed that, although RASSF5 and ITGAL are among the most important genes, their predictive ability for AAA in the GSE47472 dataset is relatively weak. In contrast, AKR1C2 and AKR1C2 demonstrated opposite trends in the GSE119717 and GSE57691 datasets. Consequently, we identified ARHGAP9 as a key diagnostic gene for AAA. The pharmacological treatment of AAA encompasses antihypertensive, lipid-lowering, and antiplatelet therapies. Consequently, we conducted an analysis of the affinity between ARHGAP9 and the drugs propranolol, lovastatin, and clopidogrel using molecular docking techniques. Our findings demonstrate that ARHGAP9 exhibits strong binding affinity with propranolol, lovastatin, and clopidogrel (Figure 7e). Furthermore, we investigated the functional role of ARHGAP9 through gene enrichment analysis, revealing its association with the NF-*κ*B, JAK-STAT, and MAPK signaling pathways. Additionally, ARHGAP9 is implicated in the regulation of stem cells and the immune system (Figure 7f).

### 3.8. ARHGAP9 Promoted the Development of AAA

**Figure 8:**
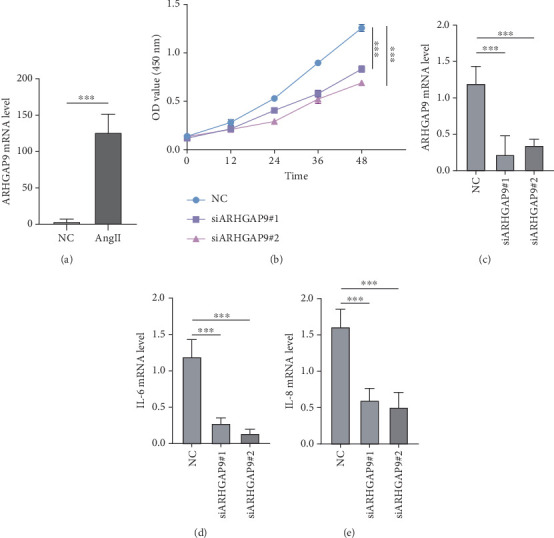
ARHGAP9 promoted the development of AAA. (a) The levels of ARHGAP9 mRNA transcripts. (b) Assessment of the inhibitory efficacy of siARHGAP9. (c) Variations in the viability of VSMCs were observed and measured prior to and following the suppression of ARHGAP9 expression in these cells. (d, e) Evaluation of the modified mRNA transcript levels of IL-6 and IL-8 in VSMCs before and after the inhibition of ARHGAP9 expression. ⁣^∗∗∗^*p* < 0.001.

Considering the crucial involvement of AngII in the development of AAA, we utilized AngII-stimulated VSMCs to recreate the AAA pathogenesis in vitro. Our investigation revealed an increase in the transcription levels of ARHGAP9 in VSMCs after AngII stimulation. This observation is consistent with predictions derived from bioinformatics analysis (Figure 8a). Importantly, the expression levels of ARHGAP9 in VSMCs post-AngII stimulation demonstrated a notable difference when compared to the control group, leading us to choose this gene for additional functional analysis. Subsequently, we designed siRNA specific to ARHGAP9 and assessed the inhibitory capabilities of two different siLCP1 pairs. The findings showed that both siARHGAP9#1 and siARHGAP9#2 had the most significant inhibitory effects (Figure 8b). After ARHGAP9 expression was suppressed, we investigated the role of ARHGAP9 on cell proliferation using CCK-8 assays, which indicated a considerable decrease in VSMC viability following ARHGAP9 suppression (Figure 8c). Additionally, we noted that inhibiting ARHGAP9 expression significantly lowered the transcription levels of IL-6 and IL-8 in AngII-stimulated VSMCs (Figure 8d,e).

## 4. Discussion

Although there have been significant improvements in the diagnostic methods for AAA, imaging continues to be the most reliable and effective approach for diagnosing AAA. Nevertheless, AAA is a particularly stealthy cardiovascular disorder that typically presents no clear clinical signs during its initial phases [26, 27]. Ongoing observation of pathological changes in patients with AAA can greatly lower the chances of aortic dissection; however, there are presently no established biomarkers for this purpose. Thus, exploring the molecular mechanisms underlying AAA may enhance our comprehension of its pathological features and developmental trajectory.

Advancements in bioinformatics have significantly contributed to the identification of disease biomarkers, particularly in the study of biomarkers associated with AAA. By integrating multiomics data and employing machine learning techniques, researchers have successfully identified several key biomarkers and potential therapeutic targets related to AAA. For instance, single-cell RNA sequencing and machine learning analysis revealed that CCR7 and CBX6 are key candidate biomarkers for AAA, showing a significant correlation with immune cell infiltration [28]. Additionally, another study utilized single-cell sequencing and machine learning to identify THBS1, HCLS1, DMXL2, and ZEB2 as biomarkers associated with AAA in macrophages. Through the THBS1-CD47 signaling pathway, it highlighted the role of macrophages in the progression of AAA [13]. In another study, researchers employed WGCNA and machine learning algorithms to identify MRAP2, PPP1R14A, and PLN as characteristic genes of AAA. These genes are closely associated with immune cell activity and the inflammatory microenvironment [29]. Furthermore, through multiomics data analysis and machine learning techniques, researchers identified CD247, CD2, and CCR7 as diagnostic biomarkers for AAA in Behcet's disease patients and developed a nomogram model for diagnosis [30]. These studies not only elucidate the molecular mechanisms of AAA but also offer new insights into the early diagnosis and treatment of the disease. By identifying and validating these biomarkers, researchers can gain a deeper understanding of the pathophysiological processes of AAA, thereby laying the groundwork for the development of new diagnostic tools and targeted therapies [31, 32]. These findings highlight the significant potential of bioinformatics in the identification and clinical application of disease biomarkers. In this research, we suggest that ARHGAP9 functions as an important diagnostic marker for AAA. Moreover, reducing ARHGAP9 levels hinders the growth potential of VSMCs. Our results also reveal that the expression of ARHGAP9 in AAA correlates significantly with the degree of immune cell infiltration.

The infiltration of immune cells is essential in understanding the pathophysiology associated with AAA. Research has demonstrated that AAA development and progression are intricately associated with the presence of various immune cells, such as neutrophils, macrophages, T cells, and B cells [33]. These immune cells contribute to inflammation and the breakdown of the extracellular matrix through the secretion of cytokines and enzymes, which in turn accelerates the degeneration of the arterial wall and the aneurysm's expansion [34]. In AAA lesions, a significant presence of macrophage infiltration is observed. Research indicates that these macrophages secrete matrix metalloproteinases, such as MMP-9, along with a range of inflammatory mediators. This activity leads to the breakdown of essential structural proteins in the arterial wall, which amplifies the inflammatory response [35]. Furthermore, macrophage infiltration is significantly associated with the aneurysm's diameter and the likelihood of rupture [36]. Apart from macrophages, the presence of T cells and B cells is also vital in the pathological mechanisms underlying AAA. Evidence indicates that the infiltration of T cells, especially CD4+ T cells, is closely related to the immune microenvironment surrounding AAA. These T cells affect AAA progression by influencing the activity of other immune cell types [37]. Meanwhile, B cells contribute to the local inflammatory regulation by establishing germinal centers and show a positive correlation with the size of the tumor and thrombus formation [38]. Moreover, the research indicated that certain chemical factors, including CXCL8 and CCL7, were elevated in AAA, facilitating immune cell infiltration and exacerbating the inflammatory response [39, 40]. These chemical factors initiated downstream signaling pathways through receptor binding, which further advanced the pathological progression of AAA. To conclude, the infiltration of immune cells and the associated molecular mechanisms are crucial in the formation and progression of AAA. A comprehensive investigation of these mechanisms may yield new targets and strategies for the early diagnosis and treatment of AAA. Our research analysis indicates that the key diagnostic genes identified are all associated with the level of immune infiltration in AAA, particularly ARHGAP9. This suggests that ARHGAP9 may play a role in regulating immune cell infiltration.

In this study, the cell experiments primarily focused on the knockdown of ARHGAP9, validating its functional impact in cell models related to AAA. However, overexpression or functional restoration experiments of ARHGAP9 have not yet been conducted, which limits a comprehensive understanding of the gene's role in regulating cellular functions. Conducting overexpression and functional restoration experiments would further confirm the biological functions of ARHGAP9 and its potential regulatory roles in signaling pathways, thereby providing stronger evidence for mechanistic studies. Future research should incorporate these experiments to refine the interpretation of ARHGAP9's effects and to establish a more robust experimental foundation for its development as a potential therapeutic target. Additionally, while current reports on ARHGAP9 are relatively limited, this study has preliminarily revealed its diagnostic value; however, exploration at the mechanistic level remains preliminary and necessitates further validation through additional molecular biology experiments.

## 5. Conclusion

This research has revealed important diagnostic genes associated with AAA by utilizing different machine learning algorithms, with ARHGAP9 emerging as the most notable. ARHGAP9 has the ability to influence the proliferation of VSMCs and the extent of immune infiltration in cases of AAA. To conclude, ARHGAP9 has the potential to function as a vital diagnostic and therapeutic marker for AAA.

## Data Availability

The data that support the findings of this study are available from the corresponding authors upon reasonable request.
